# Influence of lymph node degeneration on metastases in prostate cancer: or why we must look for a needle in a haystack

**DOI:** 10.1186/s12894-022-01167-5

**Published:** 2023-01-06

**Authors:** Daniel Gödde, Stephan Degener, Christine Walles, Rosalie Keller, Nici Markus Dreger, Katharina Graf, Friedrich-Carl von Rundstedt, Hans Michael Kvasnicka, Susanne Krege, Stephan Störkel

**Affiliations:** 1 Department of Pathology and Molecular Pathology, Helios University Hospital Wuppertal, Witten/Herdecke University, Heusnerstr. 40, 42283 Wuppertal, Germany; 2 Department of Urology, Helios University Hospital Wuppertal, Witten/Herdecke University, Heusnerstr. 40, 42283 Wuppertal, Germany; 3 Clinic for Otolaryngology, Helios Hospital Krefeld, Lutherplatz 40, 47805 Krefeld, Germany; 4 Center for Clinical Trials, Witten/Herdecke University, Alfred-Herrhausen-Str. 50, 58448 Witten, Germany; 5 Department of Urology, Evang. Kliniken Essen-Mitte, Henricistr. 92, 45136 Essen, Germany

**Keywords:** Pelvic lymph nodes, Degeneration, Prostate carcinoma, Skip metastasis, Lymph node dissection

## Abstract

**Background:**

To evaluate the incidence of lymph node degeneration and its association with nodal metastatic pattern in prostate cancer.

**Methods:**

A retrospective analysis of the submitted lymph node specimen of 390 prostatectomies in 2011 was performed. All lymph nodes were histologically re-evaluated and the degree of lymph node degeneration e.g. lipomatous atrophy, capsular and framework fibrosis, and calcifications as well as the lymph node size were recorded. Lymph node degeneration was compared in the anatomic regions of the pelvis as well as in lymph nodes with and without metastases of prostatic cancer.

**Results:**

Eighty-one of 6026 lymph nodes demonstrated metastases. Complete histologic examination with analysis of a complete cross-section was possible in 5173 lymph nodes including all lymph nodes with metastases. The incidence of lymph node degeneration was different across the various landing sites. Lymph node metastases were primarily detected in less degenerative and therefore more functional lymph nodes. In metastatic versus non-metastatic lymph nodes low lipomatous atrophy was reported in 84.0% versus 66.7% (*p* = 0.004), capsular fibrosis in 14.8% versus 35.4% (*p* < 0.001), calcifications in 35.8% versus 46.1% (*p* = 0.072) and framework fibrosis in 69.8% versus 75.3% (*p* = 0.53). Metastases were also identified more frequently in larger than in smaller lymph nodes (63.0% vs. 47.5%; *p* = 0.007).

**Conclusions:**

Degenerative changes in pelvic lymph nodes are commonly detectable but occur with variable frequency in the various nodal landing sites in the pelvis. The degree of lymph node degeneration of single lymph nodes has a significant influence on whether a lymph node is infiltrated by tumor cells and may harbour metastases.

## Background

Lymph node (LN) staging in prostatic cancer (PCa) is an essential tool in the management paradigm. The presence of LN metastasis alters the treatment concept from a local and curative therapy to an adjuvant androgen-deprivation therapy with or without radiotherapy [1, 2]. In addition, there is increasing evidence for positive therapeutic effects of pelvic lymph node dissection (PLND) [3–6]. In radical prostatectomy (RP) the extended pelvic lymph node dissection (ePLND) is recommended by the European Association of Urology (EAU) guidelines for high-risk and intermediate-risk patients with a risk for positive LN over 5% [2]. It provides relevant information for staging and prognosis which cannot be achieved by any imaging method [7]. To date, PLND still represents the most accurate and reliable staging procedure for the detection of LN invasion in PCa [8]. However, the role of PLND and its extensions remains controversial, e.g., because of associated worse intra- and peri-operative outcomes [9].

The concept of sentinel lymph node (SLN) identification and examination in PCa was introduced by Wawroschek et al. in 1999 [10]. Therefore technetium-99m colloid was applied sonographically-guided directly into the prostate 1-day prior RP with PLND. The authors were able to demonstrate a high staging accuracy for LN metastases [11–14] with a low morbidity [15]. Meanwhile, different other techniques provide a high diagnostic accuracy in LN detection, particularly the PSMA-ligand-PET imaging [16].

The presence of skip metastases in prostate cancer has been commonly explained with the multidirectional lymphatic drainage from the prostate with a high individual variability due to anatomical variations [17]. However, degeneration of lymphatic tissue accompanied by hyalinization, fibrosis and lipomatous changes is also suspected to cause skip metastases [18]. Due to these architectural disturbances, which can cause cortical gaps in the LN cortex, the LN might lose its ability to filter malignant cells or microorganisms as a result of changed intranodal homeostasis [19, 20]. The purpose of this study was to investigate the morphological changes of pelvic LN and their effect on the occurrence of metastasis.

## Methods

We retrospectively reviewed the dissected LN of 390 patients examined in our institute, who underwent RP with PLND between January and December 2011. The resected tissue of all patients was completely embedded in 4% buffered formalin fixed for histological evaluation to identify all resected LN. The study was approved by the institutional review committee (Witten/Herdecke University, No. 20/2016).

Out of 6026 detected LN, 5173 were histological reevaluated (D.G., C.W. and S.S.) on hematoxylin and eosin stained step sections for degenerative changes such as lipomatous atrophy, capsular or framework fibrosis, and calcifications as a morphological correlate for degenerative changes. Lipomatous atrophy was documented as a percentage of the incised area in stages of ≤ 30%, 31–60%, and ≥ 61%. Changes in framework fibrosis were recorded as absent, low, moderate, or severe. Capsular fibrosis and calcifications were classified as detectable or undetectable. Furthermore, the LN size (greatest diameter in millimeter) as well as the occurrence of metastases was documented. Finally, the extent of degenerative changes and LN size was compared in positive LN (with metastasis) and negative LN (without metastasis) as well for the different pelvic anatomical regions. The variables were described using absolute number and percentages. Analytic statistics was performed using the chi-square test where necessary. Results were reported as statistically significant whenever *p* < 0.05. The data were analyzed using the Statistics Package for Social Sciences version 25 (SPSS, IMB Corp, Armonk, NY, USA).

## Results

In total of 390 male patients aged 44 to 79 years (median: 68, IQR: 62–71 years) were analyzed retrospectively. In 35 patients (3.9%) aged 46 to 79 years a total of 81 metastasis out of 6026 examined LN (1.6%) were found. No age group was preferred (< 60 years: 1.5%, 61–70 years: 1.7%, 71–80 years: 1.6%; *p* = 0.601). In 15 patients only a single LN metastasis was identified, whereas in 20 patients 2–6 positive LN with metastasis could be detected. The positive LN were located in all pelvic anatomical regions but mostly in the obturator fossa in both sides. Metastasis diameter ranged between 0.5 and 18 mm (median 4, IQR 2–6 mm) occupying between 1 and 90% (median 45.5, IQR 10–70%) of the LN volume. Only six metastases demonstrated transcapsular growth. The metastatic PCa showed a broad range in both the T stage and the Gleason grading (Table 1).

**Table 1:** Metastatic prostatic carcinomas—T-Stage (UICC), Gleason Grade, and anatomical localization of metastases

T-Stage	n (%)	Gleason/ISUP	n (%)	Localization	n (%)
pT2a	1 (2.8%)	3 + 4/2	5 (14.2%)	OR	19 (23.5%)
pT2c	4 (11.4%)	4 + 3/3	8 (22.9%)	IER	4 (4.9%)
pT3a	6 (17.1%)	4 + 4/4	10 (28.6%)	IIR	8 (9.9%)
pT3b	23 (65.7%)	4 + 5/5	12 (34.3%)	OL	16 (19.8%)
pT4	1 (2.8%)			IEL	8 (9.9%)
				IIL	15 (18.5%)
				EBR/L	11 (13.5%)

*OR* Right obturator fossa, *IER* right external iliac vessels, *IIR* right iliac internal vessels, *OL* left obturator fossa, *IEL* left iliac external vessels, *IIL* left iliac internal vessels, *EBR/L* en bloc right/left without detailed allocation

**Fig. 1:**
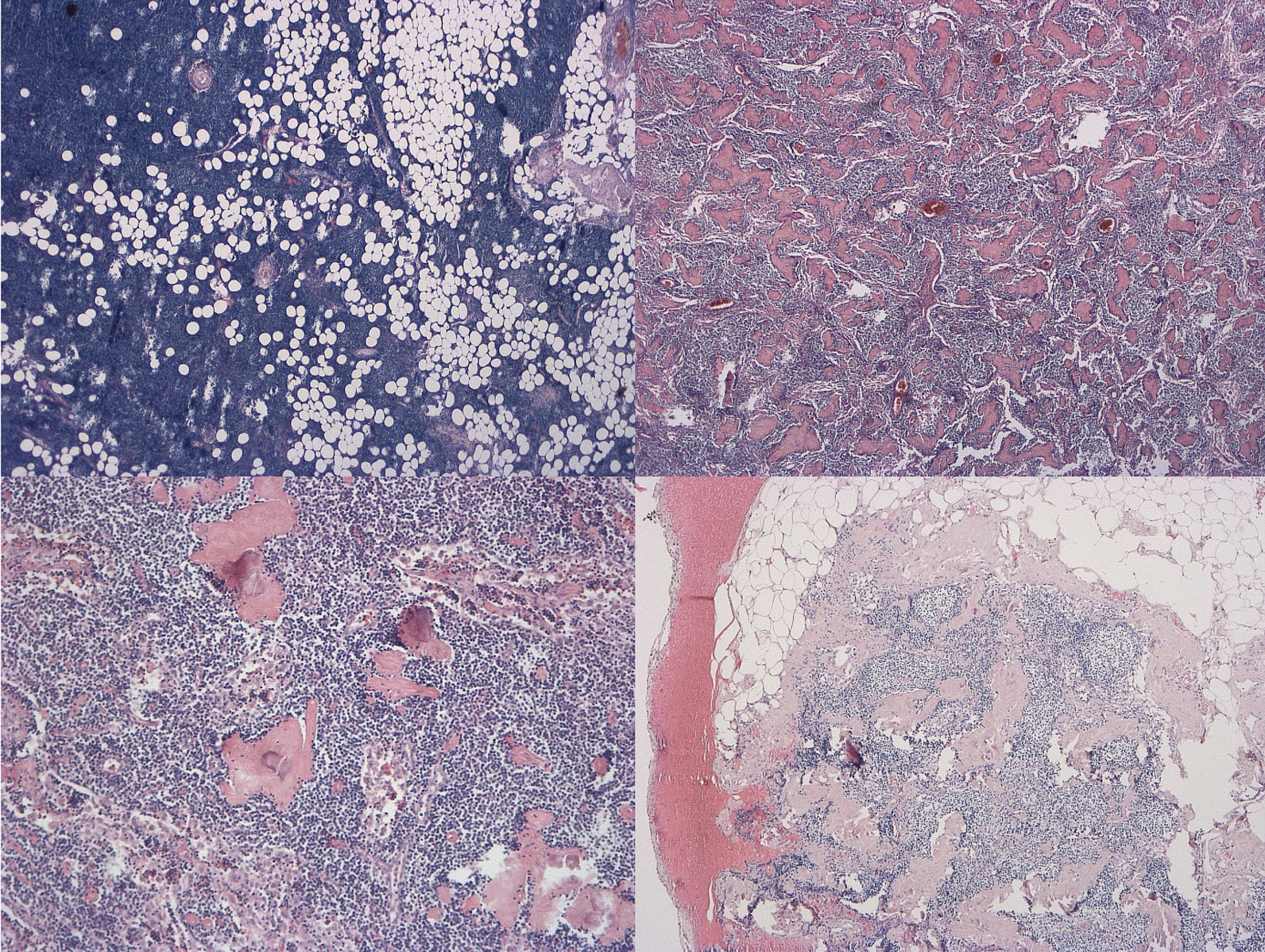
Top left—LN with lipomatous atrophy in 31–60% (H&E, 25 ×), top right—LN with high framework fibrosis (H&E, 25 ×), bottom left—LN with calcifications and moderate framework fibrosis (H&E, 100 ×), bottom right—LN with capsular fibrosis, moderate framework fibrosis and calcifications (H&E, 50 ×)

Degenerative changes in LN structure and size could be detected in all 5173 histologically examined LN. Of them, 47.8% had a minimal diameter of 10 mm, while 52.2% were smaller than 10 mm. Almost all LN showed a lipomatous atrophy in varying degrees (Fig. 1—top left): 67.0% showed a lipomatous remodeling in ≤ 30%, 13.1% in 31–60%, and 19.9% in ≥ 61% respectively. LN with every quantity of lipomatous atrophy were found in all sizes. Most of the LN showed little (23.7%) or no evidence (69.9%) of framework fibrosis with a diffuse expression form the cortex to the medulla with a nodular, later confluent enlargement of the connective tissue. A predominantly sector-shaped fibrosis with widening of the capsule appeared in 35.1% of all LN. Capsular fibrosis was more common with higher grades of framework fibrosis (Fig. 1—top right & bottom right). The association with framework fibrosis was even more pronounced for calcifications (Fig. 1—bottom left): 45.9% of the LN showed calcifications especially those LN with framework fibrosis.

Although the degenerative changes between LNs of the right and left side (LN size (*p* = 0.667), lipomatous atrophy (*p* = 0.143), capsular (*p* = 0.504) or framework fibrosis (*p* = 0.552), and calcifications (*p* = 0.143)) show no significant differences, LN of the different pelvic anatomical regions (internal and external iliac vessels and the obturator fossa) show differently pronounced, patchy, and uneven but partly significant degenerative changes (LN size (*p* = 0.012), framework fibrosis (*p* = 0.005), and calcifications (*p* = 0.032). Details are displayed in Table 2.

**Table 2:** Morphological changes in LN in different anatomical regions of the pelvis

Total 5173 LN	OR	IER	IIR	EBR	OL	IEL	IIL	EBL
	919 (17.8%)	667 (12.9%)	677 (13.1%)	318 (6.2%)	882 (17.1%)	724 (14.0%)	714 (13.8%)	272 (5.2%)
*LN size*								
< 10 mm	481 (17.8%)	338 (12.5%)	363 (13.4%)	191 (7.0%)	464 (17.2%)	386 (14.3%)	351 (13.0%)	127 (4.8%)
≥ 10 mm	438 (17.7%)	329 (13.3%)	314 (12.7%)	127 (5.1%)	418 (16.9%)	338 (13.7%)	363 (14.7%)	145 (5.9%)
								*p* = 0.012
*Lipomatous atrophy (%)*								
≤ 30	607 (17.5%)	427 (12.3%)	486 (14.0%)	242 (7.0%)	570 (16.5%)	483 (13.9%)	464 (13.4%)	185 (5.4%)
31–60	128 (18.9%)	87 (12.8%)	85 (12.5%)	26 (3.8%)	123 (18.1%)	100 (14.7%)	99 (14.6%)	30 (4.4%)
≥ 61	184 (17.8%)	153 (14.8%)	106 (10.3%)	50 (5.0%)	189 (18.3%)	141 (13.7%)	151 (14.6%)	57 (5.7%)
								*p* = 0.011
*Framework fibrosis*								
Non	637 (17.6%)	473 (13.1%)	460 (12.7%)	229 (6.4%)	624 (17.3%)	528 (14.6%)	487 (13.4%)	176 (4.9%)
Low	224 (18.3%)	157 (12.8%)	171 (14.0%)	74 (6.1%)	194 (15.8%)	145 (11.8%)	182 (14.9%)	77 (6.3%)
Moderate	54 (17.5%)	34 (11.0%)	41 (13.3%)	15 (4.8%)	57 (18.5%)	48 (15.6%)	40 (13.2%)	19 (6.1%)
High	4 (14.8%)	3 (11.1%)	5 (18.5%)	0 (0%)	7 (25.9%)	3 (11.1%)	5 (18.6%)	0 (0%)
								*p* = 0.005
*Capsular fibrosis*								
With	289 (15.9%)	243(13.4%)	264 (14.6%)	111 (6.1%)	301 (16.6%)	254 (14.0%)	270 (14.9%)	207 (6.2%)
Without	630 (18.8%)	424 (12.6%)	413 (12.3%)	82 (4.5%)	581 (17.3%)	470 (14.0%)	444 (13.2%)	190 (5.6%)
								*p* = 0.060
*Calcifications*								
With	426 (17.9%)	298 (12.5%)	315 (13.3%)	129 (5.4%)	411 (17.3%)	335 (14.1%)	334 (14.1%)	128 (5.4%)
Without	493 (17.6%)	369 (13.2%)	362 (12.9%)	189 (6.7%)	471 (16.8%)	389 (13.9%)	380 (13.6%)	144 (5.3%)
								*p* = 0.032

No significant differences in the occurrence of LN metastases between right and left side were found (right: 37, left 44; *p* = 0.262). Concerning the positive lymph nodes, 63.0% measured more than 10 mm in diameter (vs. 47.5% in negative LN) with an overall LN size ranging from 3 to 50 mm in diameter (median 11, IQR 8–17 mm). 9 LN with metastases were less than 5 mm in size. The structural changes of LN architecture in positive LN were less pronounced in comparison with negative LN: 84.0% of LN with metastasis showed only little lipomatous atrophy (vs. 66.7% in negative LN, *p* = 0.004) and a capsular fibrosis was seen only in 14.8% versus 35.4% respectively (*p* < 0.001). Although the results for calcifications were not statistically significant, the absolute numbers show a tendency towards less degenerative changes in positive LN. No significant difference could be shown for framework fibrosis (Table 3).

**Table 3:** Morphological changes in LN with and without metastases

	Metastasized LN	Non-metastasized LN
*Lymph node size*		
< 10 mm	30 (37.0%)	2671 (52.5%)
≥ 10 mm	51 (63.0%)	2421 (47.5%)
		*p* = 0.007
*Lipomatous atrophy (%)*		
≤ 30	68 (84.0%)	3396 (66.7%)
31–60	7 (8.6%)	671 (13.2%)
≥ 61	6 (7.4%)	1025 (20.1%)
		*p* = 0.004
*Framework fibrosis*		
Non	61 (75.3%)	3553 (69.8%)
Low	15 (18.5%)	1209 (23.7%)
Moderate	4 (4.9%)	304 (6.0%)
High	1 (1.2%)	26 (0.5%)
		*p* = 0.53
*Capsular fibrosis*		
With	12 (14.8%)	1802 (35.4%)
Without	69 (85.2%)	3290 (64.6%)
		*p* < 0.001
*Calcifications*		
With	29 (35.8%)	2347 (46.1%)
Without	52 (64.2%)	2745 (53.9%)
		*p* = 0.072

## Discussion

The staging of pelvic LN in PCa allows a statement with regards to prognosis and treatment options and therefore LN dissection in PCa is recommended for intermediate and high-risk patients [21]. Lymphatic drainage from the prostate has high individual variability, and direct drainage outside the pelvic area is observed rarely [17, 22]. Despite advances in imaging modalities the gold standard in LN is the histological evaluation of an ePLND [23, 24]. Due to a up to three-fold higher complication rates compared with a limited pelvic LN dissection [25] the role of ePLND remains controversial, especially since PLND failed to improve oncological outcomes, including survival [9].

Because of prostate specific antigen relapse, symptomatic progression and tumor related death are significantly affected by the number of positive LN [6, 26, 27] the detection rate for positive LN must be optimized. To achieve this goal, a better understanding of the lymphogenic metastatic behavior of PCa in pelvic LN is required. The present series is the first histological analysis based on a large cohort that focuses on degenerative changes in pelvic LN in connection with pelvic LN localization and the occurrence of metastases in PCa. Our histological evaluation revealed significant morphological differences between pelvic LN in different anatomical localizations as well in LN with or without metastases of prostatic cancer.

Changes in LN morphology have been described in connection with the aging process as well as with chronic inflammatory diseases. Pan et al. [28] proposed a generating-degenerating circle of LN affecting all compartments including the lymphatic tissue, the medulla, and the architecture. Sato et al. described similar changes in LN of older patients with formation of gaps and fragmentation of the superficial cortex. These degenerative changes are less pronounced in central like gastric LN than in peripheral like para-aortic and pelvic LN [19]. Furthermore, lipomatous atrophy is described in other studies as a basin-specific degenerative change of peripheral LN usually subjected to little antigenic stimulation [18, 20]. We were able to prove, that degenerative changes in pelvic LN are always detectable in older male patients but occur inconsistently within the different anatomical locations of the pelvis. These findings suggest the idea that degenerative changes may be somewhat random in their occurrence. The grade of the degenerative changes is associated with the anatomical localization of the LN and may be caused by its exposure to more or less antigenic stimulation. Degenerative changes may have an effect on the metastatic pattern found in lymphadenectomy specimens [28] and are possibly caused by the altered immune status in the elderly [20, 29, 30].

## Conclusions

Degenerative changes in pelvic LN are commonly detectable but occur with variable frequency in the various nodal landing sites in the pelvis. We were able to demonstrate that metastases are predominantly detected in larger and less degeneratively altered LN in the pelvis. The degree of LN degeneration of single LN has a significant influence on whether a LN is infiltrated by tumor cells and may harbour metastases. This, in addition to multidirectional lymphatic drainage, is an additional explanation for the occurrence of skip metastases in prostate cancer, resulting in an unpredictable metastasis pattern.

## Data Availability

The datasets used and/or analyzed during the current study are available from the corresponding author on reasonable request.

## Abbreviations

LN: Lymph node
PCa: Prostatic cancer
PLND: Pelvic lymph node dissection
RP: Radical prostatectomy
ePLND: Extended pelvic lymph node dissection
EAU: European Association of Urology
SLN: Sentinel lymph node
IQR: Interquartile range
OR: Right obturator fossa
IER: Right external iliac vessels
IIR: Right iliac internal vessels
EBR: En bloc right
OL: Left obturator fossa
IEL: Left iliac external vessels
IIL: Left iliac internal vessels
EBL: En bloc left
